# Nonadiabatic Wave Packet Dynamics with Ab Initio Cavity-Born-Oppenheimer Potential Energy Surfaces

**DOI:** 10.1021/acs.jctc.2c01154

**Published:** 2023-01-10

**Authors:** Thomas Schnappinger, Markus Kowalewski

**Affiliations:** Department of Physics, Stockholm University, AlbaNova University Center, SE-106 91Stockholm, Sweden

## Abstract

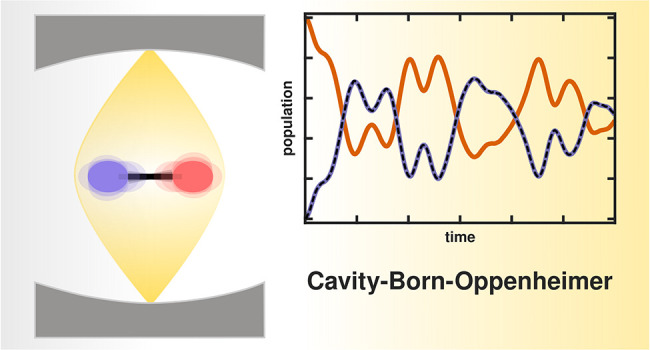

Strong coupling of molecules with quantized electromagnetic fields can reshape their potential energy surfaces by forming dressed states. In such a scenario, it is possible to manipulate the dynamics of the molecule and open new photochemical reaction pathways. A theoretical approach to describe such coupled molecular-photon systems is the Cavity-Born-Oppenheimer (CBO) approximation. Similarly to the standard Born-Oppenheimer (BO) approximation, the system is partitioned and the electronic part of the system is treated quantum mechanically. This separation leads to CBO surfaces that depend on both nuclear and photonic coordinates. In this work, we demonstrated, for two molecular examples, how the concept of the CBO approximation can be used to perform nonadiabatic wave packet dynamics of a coupled molecular-cavity system. The light-matter interaction is incorporated in the CBO surfaces and the associated nonadiabatic coupling elements. We show that molecular and cavity contributions can be treated on the same numerical footing. This approach gives a new perspective on the description of light-matter coupling in molecular systems.

## I Introduction

Within the last years, the topic of strong light-matter interaction has gained a lot of interest in the fields of chemistry and material science. In experiments, it is observed that molecules, when placed in an optical cavity,^1−9^ show modified reaction rates and altered optical properties. On the basis of the well-understood Jaynes-Cummings model,^10^ the underlying effect is explained by the formation of hybrid light-matter states, also called polaritons or dressed states. In a molecular system, this basic concept of polaritons still holds, but the presence of nuclear degrees of freedom makes the description considerably more complex. Depending on whether the quantized cavity modes are coupled via their characteristic frequency to electronic or vibrational degrees of freedom of the molecule, the situation is described as electronic strong coupling (ESC) or vibrational strong coupling (VSC), respectively. For ESC, the photochemistry, including charge transfer processes and electronic spectroscopy, is affected.^11−23^ In the VSC regime, the chemistry of a single electronic state (mostly the ground state) and its vibrational spectroscopy are influenced by the cavity interaction.^5,9,24−28^ Generally, ESC and VSC can open up a fundamentally new approach for altering chemical reactivity.

As a result of the high complexity of the molecules themselves, the Jaynes-Cummings model, which has been developed for two-level atoms coupled to a cavity, is only an approximation. Therefore, existing models have been extended, or new theoretical methods have been developed at the border of quantum-chemical ab initio methods and quantum optics. To describe a coupled molecule-cavity system in the ESC regime, extended versions of the Jaynes-Cummings model are often applied in the literature.^18−20,23,29,30^ In these models, the coupled molecular-photon system can be formulated on the basis of photon states, also called Fock states,^29−32^ or by using photon displacement coordinates.^18−20^ In both representations, the field-free expectation values of the molecular dipole moments are used to calculate the interaction of the molecule and the photon field. The obtained interaction terms, describing the cavity-induced coupling between surfaces, are added as coupling to the bare molecular Hamiltonian. If the coupling is smaller than the transition energy, these models can also be used to describe VSC.^27^ In recent years, electronic structure methods^33−37^ have been developed to obtain an ab initio approach to calculate the interaction between the cavity and the molecule on the basis of Fock states. The Cavity-Born-Oppenheimer (CBO) approximation offers an alternative strategy to describe a coupled light-matter system in an ab initio fashion in the basis of photon displacement coordinates. In the CBO approximation, the electronic and nuclear-photonic degrees of freedom can be separated^38−40^ and the resulting electronic problem can be solved using standard electronic structure methods. Electronic energies obtained for different nuclear configurations and photon displacements define the potential energy surface (PES) or, more precisely, the cavity potential energy surface (cPES). This cPES automatically incorporates all interactions between the molecule and the cavity mode. Since the CBO approximation includes the interaction between electrons and the electric field into the electronic structure calculation, the results can be expected to be valid for the strong coupling regime and the ultrastrong coupling (USC) regime.^38,39,41^ The CBO ansatz has been mostly implemented for different variations of density functional theory.^38,40,42,43^ Generally, CBO is a good choice for the description of a coupled molecule-cavity system in the VSC regime.^28,44−46^

In this paper, we investigate how the concept of CBO can be applied to perform nonadiabatic wave packet dynamics in a coupled molecule-cavity system in the ESC regime. Since the description of a ESC system requires multiple electronic states, respectively cPESs, one must go beyond the CBO to describe field-induced transitions between electronic states. The transitions between cPESs states are then mediated by nonadiabatic coupling elements (NACEs) and become nonadiabatic processes, analogous to nonadiabatic transitions caused by avoided crossings and conical intersections (CoIns). We combine the CBO approximation with the complete active space self-consistent field (CASSCF) method and the multiconfiguration reference configuration interaction (MRCI) method to ensure an adequate description of the cavity-induced effects on ground- and excited-state energies, as well as the nonadiabatic couplings. The obtained cPESs and couplings are then used to perform nonadiabatic quantum wave packet dynamics in the coupled molecule-cavity system. In principle, both the ESC and VSC regimes can be described in this CBO ansatz. The VSC is directly included in the cPESs, and the ESC features, namely, the cavity-induced coupling between different electronic states, take the form of NACEs. Using the cPESs and NACEs to perform nonadiabatic wave packet dynamics or semiclassical surface hopping could be an interesting strategy to simulate dynamics in hybrid light-matter systems.

In the first part, the theory of the CBO approximation and the concept of the nonadiabatic couplings are explained. After setting the stage, we discuss the methods used to calculate cPESs and couplings, as well as how the wave packet dynamics is performed. As a proof of principle, we present two numerical examples of real molecular systems. The first example is the dynamics of the first two bound stats of MgH^+^, and the second test case is the photodissociation of LiF. In the MgH^+^ system, all nonadiabatic effects are induced by the cavity, and in LiF, the intrinsic avoided crossing is strongly modified by the cavity interaction. In both examples, the simulations are initiated by assuming a delta pulse excitation and the resulting nonadiabatic wave packet dynamics based on CBO is compared with the one simulated with the extended molecular Jaynes-Cummings model (EJCM). For comparison, the EJCM ansatz in this work is formulated in photon displacement coordinates, and the interaction is calculated using field-free expectation values. In addition, dipole self-energy (DSE) terms are approximately included in the simulation, as they have been shown to be important in the description of the light-matter interaction in the USC regime and the VSC regime.^19,20,26,47,48^

## II Theory

### II.A General Correlated Electron-Nuclear-Photon Systems

We consider a molecule coupled to a single cavity mode, and the corresponding Hamiltonian (eq 1) in the length gauge and dipole approximation is constructed from the respective subsystems and their interaction. Atomic units are used throughout the paper, unless otherwise indicated.
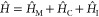
1Here, the molecular Hamiltonian *Ĥ*_M_ is formulated in the spirit of the standard BO approximation consisting of the nuclear kinetic energy operator *T̂*_N_ and the electronic Hamiltonian *Ĥ*_e_.
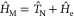
2All Coulombic interactions (electron-electron (*V̂*_e,e_), nuclear-nuclear (*V̂*_N,N_), and electron-nuclear (*V̂*_e,N_)) and the kinetic energy of the electrons are included in *Ĥ*_e_:
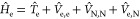
3The harmonic, single-cavity mode Hamiltonian *Ĥ*_C_ is formulated using photon displacement coordinates:^18,49,50^
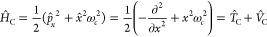
4where ω_*c*_ is the characteristic frequency of the cavity mode. *x* and *p̂*_*x*_ are the photon displacement coordinate and its conjugate momentum operator, respectively. Note that the photon displacement coordinate is treated in a manner similar to the nuclear coordinates: the single-cavity mode is described by kinetic and potential energy operators (*T̂*_C_ and *V̂*_C_). Nevertheless *x* and *p̂*_*x*_ can be expressed in terms of the bosonic photon creation and annihilation operators (*a* and *a*^†^):^18^
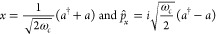
5The third term in eq 1, *Ĥ*_*I*_, describes the coupling between the photon field and the electrons and nuclei of the molecule in the dipole approximation^40,41,47^
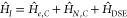
6The first two terms *Ĥ*_*e*,C_ and *Ĥ*_*N*,C_ resemble the bare light–matter interaction:
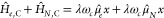
7Here, μ̂_e_ and μ̂_N_ are the electronic and nuclear parts of the dipole moment operator. The coupling parameter λ^40,42^ can be directly connected to the cavity vacuum electric field strength (ϵ_*c*_):
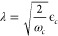
8The coupling parameter (λ) depends on the volume of the cavity mode and the dielectric constant of the material inside the cavity. Explicitly, we consider only a single molecule here and assume a nonlossy cavity. Furthermore, we treat λ as a tunable coupling parameter in this work. The third contribution to *Ĥ*_*I*_ is the (DSE) term *Ĥ*_DSE_, which must be taken in to account at larger coupling strengths.^19,26,48^ Again, this contribution can be separated into a nuclear component *Ĥ*_DSE_^*N*^ and terms containing electronic contributions (dipole moment operator μ̂_*e*_ and squared dipole moment operator μ̂_*e*_^2^).
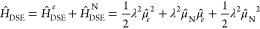
9Based on eq 1, two possible ways are discussed in this paper to perform nonadiabatic wave packet dynamics in a coupled molecular-cavity system. The main difference between the two methods is how the interaction between the molecule and the cavity photon field is included in the simulation.

### II.B Extended Molecular Jaynes-Cummings Model

The first method is an extended version of the well-known Jaynes-Cummings model^10^ and is used as a reference for the results of CBO ansatz. We term the model ”extended” since it adapted to a molecular system and contains both the counter-rotating terms in the coupling Hamiltonian and the DSE terms.^18−20^ In this model, all molecular properties such as PESs and dipole moments are calculated without any cavity photon-field interaction and following the standard BO approximation by solving the time-independent electronic Schrödinger equation:
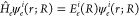
10By solving it for different nuclear configurations, the resulting eigenvalues, *E*_*i*_(*R*), define the PES of a given electronic state *i* characterized by the electronic eigenfunctions ψ_*e*_^*i*^(*r*; *R*). The single-cavity mode Hamiltonian *Ĥ*_C_ is formulated according to eq 4, and the total EJCM Hamiltonian has the following form:
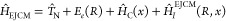
11The coupling Hamiltonian *Ĥ*_*I*_^EJCM^ within EJCM is defined as follows:
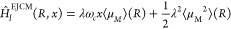
12It contains a linear light-matter interaction term, as well as a quadratic DSE term, which are calculated with the field-free expectation values of the dipole operator ⟨μ_M_⟩(*R*) and the squared dipole operator ⟨μ_M_^2^⟩(*R*), respectively. To describe nonadiabatic processes within the EJCM, the bare molecular nonadiabatic or diabatic couplings are used. Additional contributions of the cavity interaction are determined by the field-free expectation values of the transition dipole moments and their squared versions. In principle, the interaction of the molecule and the photon field in EJCM is not taken into account in an ab initio procedure, but rather as a correction to the bare molecular system.

### II.C Cavity-Born-Oppenheimer Approximation

The main focus of this work is on the second approach, namely, the CBO approximation.^38−40^ Generally, the CBO approximation is based on the idea that the total correlated electron-nuclear-photon system can be separated in two parts: an electronic subsystem and a nuclear-photonic contribution. The total wave function Ψ_tot_(*r*, *R*, *x*) is formulated as a sum of product states
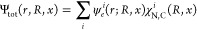
13with the electronic wave functions ψ_*e*_^*i*^(*r*; *R*, *x*) and the combined nuclear-photonic wave functions χ_N,C_^*i*^(*R*, *x*) of the electronic state *i*. The electronic wave functions depend on the electronic coordinates *r* and have only parametric dependency on the nuclear coordinates *R* and photon displacement coordinate *x*. Following the arguments of the standard BO approximation, the electrons can “quasi-instantaneously” adapt to changes of the nuclear coordinates, since the nuclei are much “slower” than the electrons. Regarding the photon field, a very similar argument can be made. If the photon field changes only ”slowly” over time, the electrons can adapt quasi-instantaneously to these ”slow” changes.^38,39^ Or, in other words, the action of the operators *T̂*_N_ and *T̂*_C_ on the electronic wave functions is equal to zero. These assumptions allow for the separation of the electronic subsystem and, thus, the formulation of an electronic Hamiltonian *Ĥ*_*e*_^CBO^ within CBO.
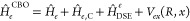
14*Ĥ*_*e*_ is the electronic Hamiltonian as in the standard BO approximation (see eq 3). The coupling between the photon field and the electrons is described by the two operators *Ĥ*_*e*,C_ and *Ĥ*_DSE_^*e*^ (eqs 7 and 9). All contributions of the nuclei and of the cavity are summed up in a scalar potential *V*_*ex*_(*R*, *x*) for a given combined nuclear and photon configuration.
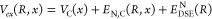
15By solving the electronic Schrödinger equation under the CBO approximation (eq 16) for different nuclear and photon configurations, the resulting eigenvalues *E*_*e*_^*i*^(*R*, *x*) define the cPES of a given electronic state *i*.
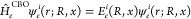
16This leads to the total CBO Hamiltonian:
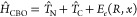
17

### II.D Beyond the Cavity-Born-Oppenheimer Approximation

Just like in a standard BO description, there are situations in the CBO approximation where the cPESs get close in energy and the adiabatic decoupling breaks down. Therefore, nonadiabatic coupling terms must be taken into account. Moreover, the nonadiabatic couplings are required to describe transitions between electronic states even when the CBO states are well-separated. In the CBO description, not only *T̂*_N_ but also *T̂*_C_ can affect the electronic subsystem, leading to two sets of coupling elements.
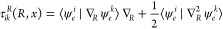
18
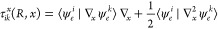
19Both τ_*ik*_^*R*^(*R*, *x*) and τ_*ik*_^*x*^(*R*, *x*) contain derivative coupling terms and scalar coupling terms. The elements τ_*ik*_^*R*^(*R*, *x*) are the nonadiabatic coupling terms coupling states *i* and *k*. Besides the dependency on *x*, they are comparable to the coupling elements that are required to describe dynamics beyond the standard BO approximation. The second type of elements τ_*ik*_^*x*^(*R*, *x*) describes the coupling between cPESs solely induced by the interaction of the molecule with the cavity. These elements are responsible for the cavity-driven population transfer between electronic states in the ESC regime. In contrast to the EJCM model, the bare molecular couplings and the cavity-induced couplings are subjected to completely identical treatments here. Consequently, it is possible to directly describe the modification of a molecular CoIns and the newly created cavity/light-induced CoIns. In both cases, the NACEs diverge to ±∞, which can lead to numerical problems. Following the established ideas of nonadiabatic dynamics,^51−54^ these difficulties can be avoided by transformation from the adiabatic representation to the diabatic representation. In a two-state example, the diabatic electronic wave functions (ϕ_*e*_^1^(*r*; *R*, *x*), ϕ_*e*_^2^(*r*; *R*, *x*)) can be determined as a linear combination of the adiabatic electronic wave functions:
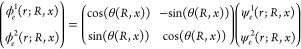
20The mixing angle θ(*R*, *x*) between the adiabatic wave functions can be defined in an interval of [0, π] and is obtained by requiring NACEs τ_12_^*R*^ and τ_12_^*x*^ to vanish. The resulting diabatic coupling is described by the potential-like scalar quantity, which unifies both the bare molecular couplings and the cavity-induced couplings.

## III Methods

**Figure 1:**
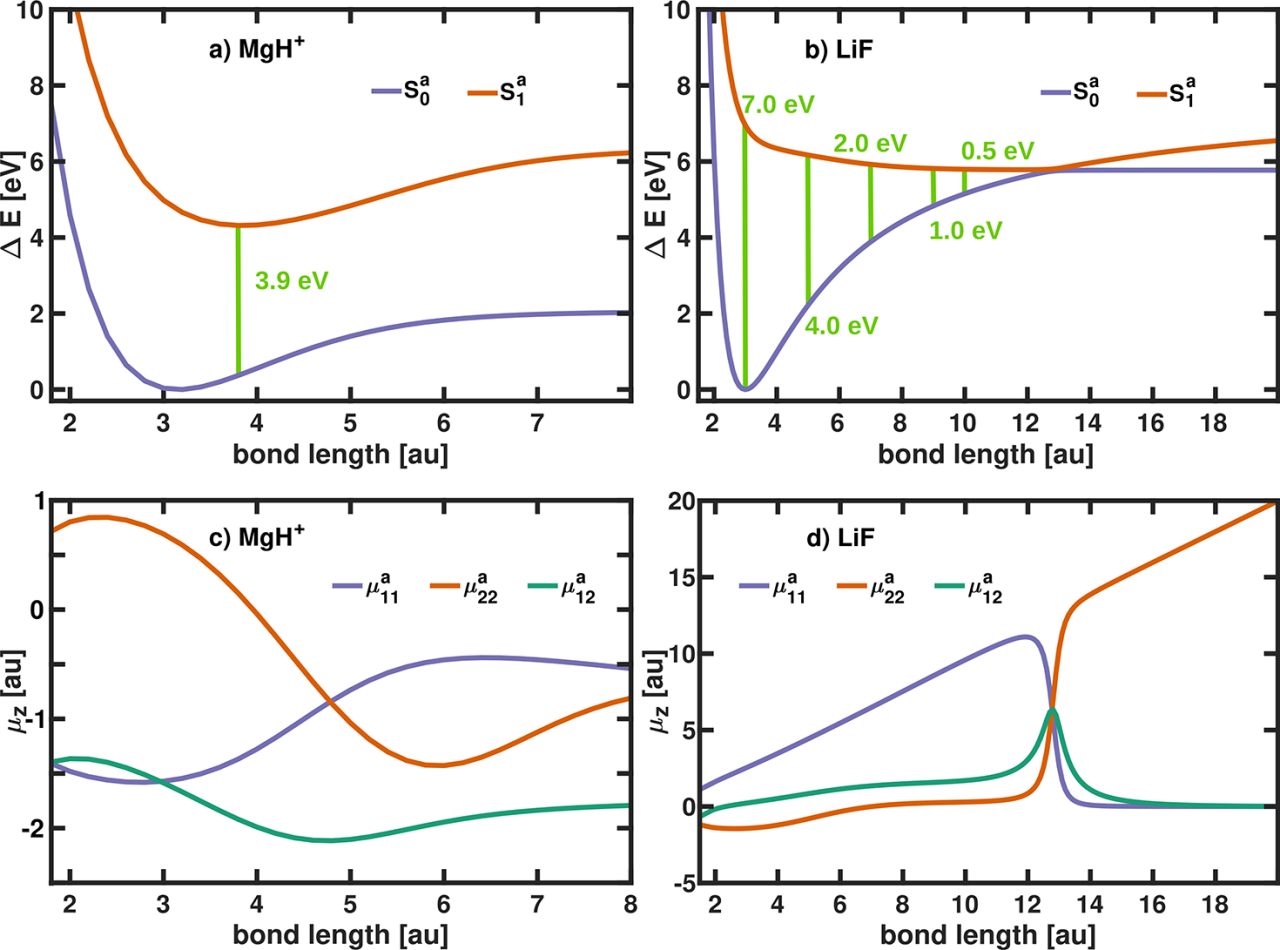
Bare ground (*S*_0_^*a*^) and excited (*S*_1_^*a*^) electronic potential energy surfaces of (a) MgH^+^ and (b) LiF in the adiabatic representation. Light green lines indicate the cavity mode frequencies ω_*c*_ considered in the current study. Corresponding permanent and transition dipole moments (μ_11_^*a*^, μ_22_^*a*^, and μ_12_^*a*^) along the molecular bond (*z*-axis) of (c) MgH^+^ and (d) LiF.

After introducing the theoretical concepts, we want to demonstrate how nonadiabatic wave packet dynamics can be performed within the CBO approximation and EJCM. Our two showcase systems are MgH^+^ and LiF coupled to an optical cavity. In both cases, we restrict our study to the dynamics in the first two electronic states. All electronic structure calculations are performed with the program package MOLPRO, version 2020.1,^55^ at the complete active space self-consistent field (CASSCF) and the multiconfiguration reference configuration interaction (MRCI) level of theory.^56−59^ All calculations were performed in a reproducible environment, using the Nix package manager, together with NixOS-QChem^60^ (commit 8bf1cc07) and Nixpkgs (nixpkgs, 21.05, commit b2f87e00). Following the CBO approximation, the two operators *Ĥ*_*e*,C_ and *Ĥ*_DSE_^*e*^ must be included in the electronic Schrödinger equation (eq 16). For this purpose, the matrix facility (MATROP) of MOLPRO is used, which allows us to access and modify the necessary operators on the basis of atomic orbitals. Since *Ĥ*_*e*,C_ is a one-electron operator, it can be expressed in terms of the dipole moment operation that already exists. Consequently, *Ĥ*_*e*,C_ takes the following form:
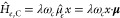
21with **μ** being the integrals of the electric dipole moment. In this work, we approximate the electronic DSE operator by taking into account only the one-electron contributions and neglecting the two-electron terms. In this one-electron approximation, *Ĥ*_DSE_^*e*^ takes the following form:
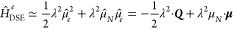
22with ***Q*** being the electronic part of the second-moment integrals. Here, only the diagonal elements (*Q*_*xx*_, *Q*_*yy*_, *Q*_*zz*_) of the second-moment tensor are required for the calculation of the DSE. Both operators *Ĥ*_*e*,C_ and *Ĥ*_DSE_^*e*^ are added to the full one-electron Hamiltonian used in the CASSCF/MRCI calculations. After convergence, the scalar potential *V*_*ex*_(*R*, *x*) is added to the electronic energy. The derivative coupling terms are computed by finite differences of the electronic wave functions by applying the second-order algorithm implemented in MOLPRO.^55^ The diabatic electronic states at the MRCI level of theory were constructed using the orbital-based quasi-diabatization procedure implemented in MOLPRO.^61,62^ This procedure relies on minimization of the derivative couplings by employing the condition of configurational smoothness of the diabatic states. Starting from a suitable reference geometry, in our case, the Franck–Condon (FC) point, the diabatic electronic wave functions at neighboring geometries are determined by maximizing the overlap with the reference electronic wave function, using a unitary transformation matrix. For MgH^+^, an established^32,63,64^ active space of two electrons in 11 orbitals CASSCF(2/11), in combination with the ROOS basis set,^65,66^ is applied. The active space in the LiF case is CASSCF(6/12) in combination with the aug-cc-pVTZ basis set.^67^ The adiabatic PESs and permanent and transition dipole moments for the first two adiabatic electronic states of the bare MgH^+^ and LiF molecule are shown in Figure 1.

The first two PESs of the bare MgH^+^, shown in Figure 1a, are both representing bound states and do not form any avoided crossing. Both states exhibit a permanent dipole moment and a transition dipole moment along the *z*-axis (Figure 1c) and can be coupled to the quantized light field of a cavity that is resonant with the electronic excitation energy. Since the ground state (*S*_0_^*a*^) and the excited state (*S*_1_^*a*^) are well-separated, population transfer can only happen via the interaction with the cavity. In contrast, the adiabatic electronic states of the field-free LiF experiences an avoided crossing at a bond length of ∼13 a.u., which leads to dissociation into neutral products through the ground state *S*_0_^*a*^ (Figure 1b). In the avoided crossing region, the permanent dipole moments changes suddenly and the transition dipole moments peaks (Figure 1d). The second example of LiF is used to study the effect of cavity coupling on the nonadiabatic dynamics. All studied cavity frequencies ω_*c*_ are shown in Table 1 and are indicated as light green lines in Figures 1a and b.

**Table 1:** Studied Cavity Frequencies (ω_*c*_) and Minimum and Maximum Values of the Bond Length (*r*) and the Photon Displacement Coordinate (*x*)

	MgH^+^	LiF				
**Cavity Frequency**						
ω_*c*_ [eV]	3.9	7.0	4.0	2.0	1.0	0.5
**Minimum and Maximum Bond Lengths**						
*r*_min_ [a.u.]	1.80	1.80	1.80	1.80	1.80	1.80
*r*_max_ [a.u.]	14.00	18.00	18.00	20.00	20.00	25.00
**Minimum and Maximum Photon Displacement Coordinates**						
*x*_min_ [a.u.]	–12.00	–15.00	–15.00	–20.00	–25.00	–45.00
*x*_max_ [a.u.]	12.00	15.00	15.00	20.00	25.00	45.00

The nonadiabatic wave packet dynamics of the cavity coupled systems have been simulated by numerically solving the time-dependent Schrödinger equation of the nuclear-photonic subsystem with the Hamiltonian given in eqs 11 and 17. The bare molecular Hamiltonian (eq 2) was used in the field-free case. The wave packet is propagated according to the Arnoldi propagation scheme^68^ on the cPESs, which are represented by a one-dimensional, respectively, two-dimensional numerical grid with 256 points along the bond length *R* and 128 points along the photon displacement coordinate *x*. The respective minimum and maximum coordinate values can be taken from Table 1. The nuclear wave packets have evolved for 100 fs (MgH^+^) and 200 fs (LiF) with a time step of 24 attoseconds, using our in-house quantum dynamics code (QDng). For the LiF case, the perfect matched layer (PML) has been placed on the edges of the bond length coordinate, as the absorbing boundary condition.^69^ For the analysis, all absorbed parts of the wave packet are counted as dissociated. To initiate the dynamics simulation in the first excited electronic state, we assume a delta pulse excitation. This is realized by vertically placing the first vibrational eigenfunction of the ground electronic state on the excited state surface. The vibrational eigenfunctions of the ground state potential are obtained by employing the imaginary time propagation method.^70^ The nonadiabatic coupling terms are included in the simulation, as described elsewhere.^53,71,72^ Two quantities are used to analyze the nonadiabatic dynamics in the coupled molecule-cavity systems. The first one is the population in the two electronic states of the molecular system and the second one is the number of photons in the cavity. The latter quantity is calculated as the expectation value of the photon number operator:^50^
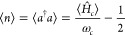
23

## IV Results and Discussion

### IV.A MgH^+^

**Figure 2:**
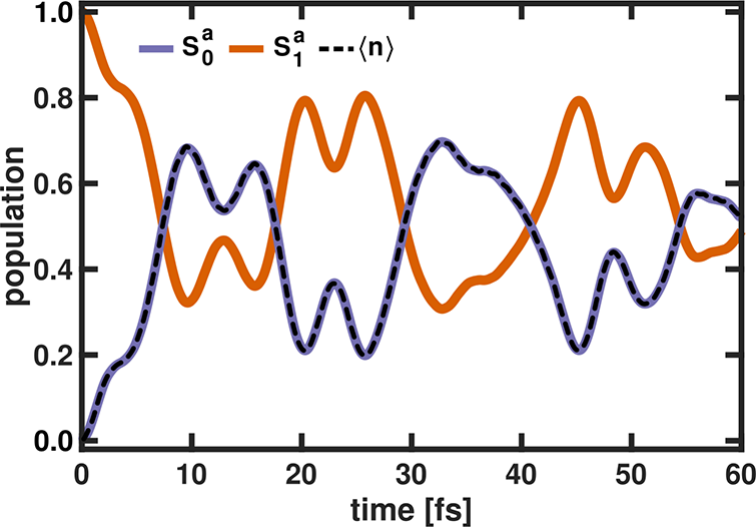
Time-dependent population in the two electronic states (*S*_0_^*a*^ and *S*_1_^*a*^) in MgH^+^ and the photon number ⟨*n*⟩ (black dotted line) for ω_*c*_ = 3.9 eV and ϵ_*c*_ = 2.0 V nm^–1^ calculated in the CBO ansatz.

MgH^+^ is used to study the capabilities of performing nonadiabatic wave packet dynamics in the CBO formalism. In this case, we investigate the importance of the DSE terms in the dynamics in the CBO description and compare it with the results obtained using the EJCM ansatz. The bare MgH^+^ dynamics in the first excited state is simply characterized by an oscillation of the nuclear wave packet and no population transfer is observed. The chosen cavity frequencies ω_*c*_ = 3.9 eV is resonant with the energy gap between the first two electronic states at the *S*_1_^*a*^ minimum (see Figure 1a, light green line). Figure 2 shows the temporal evolution of the population in the two electronic states and the expectation value of the photon number for ϵ_*c*_ = 2.0 V nm^–1^ calculated in the CBO ansatz.

For the shown coupling strength, the time-dependent populations show a quite complicated oscillating pattern. With increasing simulation time, the populations of *S*_0_^*a*^ and *S*_1_^*a*^ are approaching 0.5. Two oscillation periods can be clearly observed: a slow one of ∼25 fs and a faster one close to 10 fs. In the field-free situation, the vibrational period of the ground state is 20 fs and that of the excited state is 30 fs.^73^ Once MgH^+^ interacts with the cavity mode, the two states are strongly coupled in a nonadiabatic way and the motion of the nuclear-photonic wave packet incorporates both field-free periods in a nontrivial way. For instance, the period of 25 fs is approximately the average of the two field-free values and the observed fast period corresponds well to the difference of the two vibrational periods. The interaction with the cavity is the only source of population transfer between the states and thus the two states are fully decoupled in the bare molecule. The population transfer in the coupled MgH^+^ system corresponds to a transfer between the photonic and molecular subsystems. Therefore, the photon number ⟨*n*⟩ shows the same behavior as the temporal evolution of the ground state population and directly reflects the molecular motion. As previously suggested in the literature,^74,75^ the detection of the cavity photon could be used, in principle, as an internal probe of the molecular dynamics.

**Figure 3:**
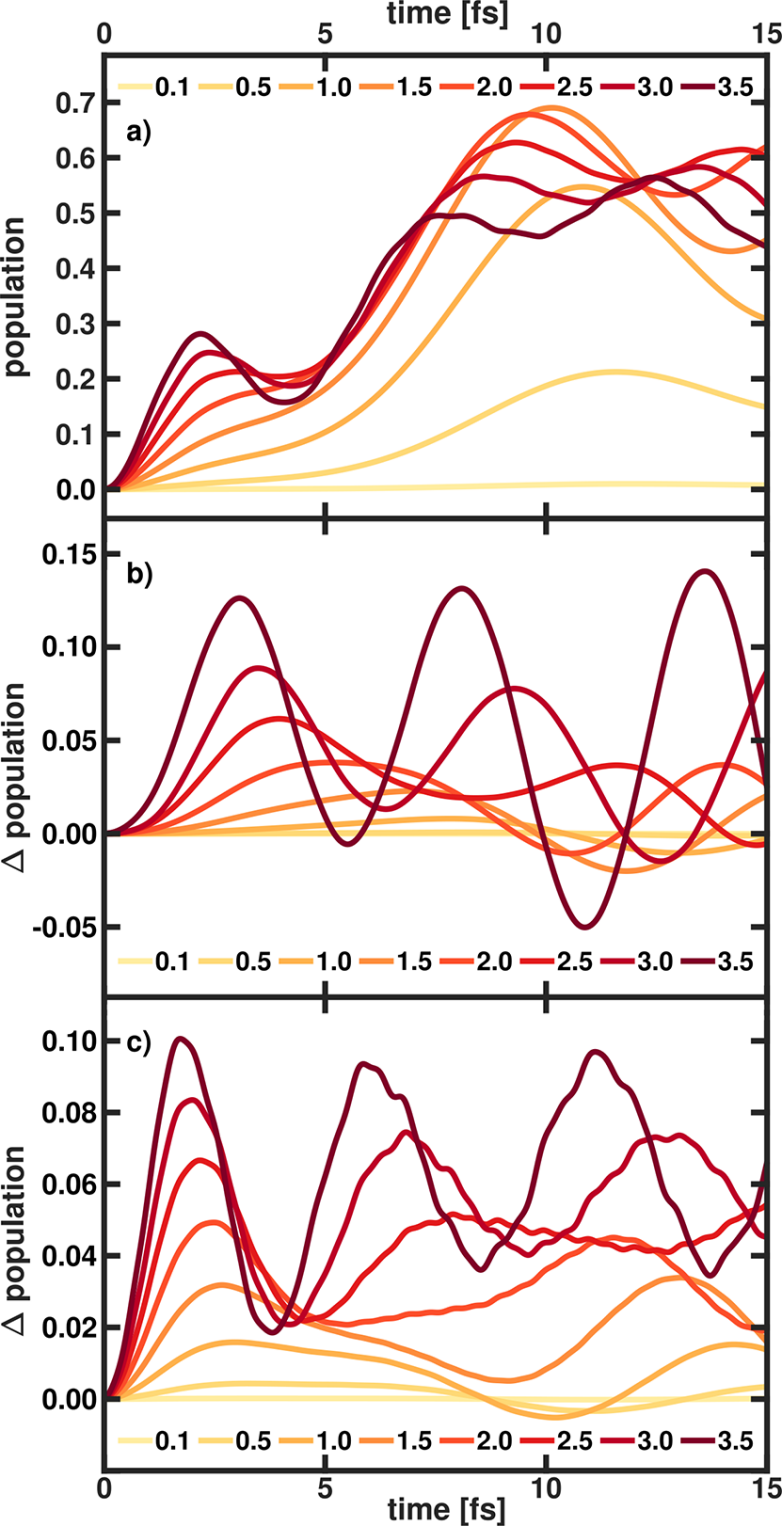
(a) Time-dependent population in the ground state *S*_0_^*a*^ of MgH^+^ obtained using CBO with DSE terms. (b) Difference in the *S*_0_^*a*^ population with and without DSE terms included in the CBO dynamics. (c) Difference in the *S*_0_^*a*^ population between the CBO dynamics and the EJCM dynamics both with DSE terms included. All simulation were performed with ω_*c*_ = 3.9 eV and the cavity field strength ϵ_*c*_ was increased from 0.1 V nm^–1^ to 3.5 V nm^–1^ (from lightest color to darkest color).

The influence of the coupling strength on the dynamics of the coupled MgH^+^ system is studied by varying the cavity field strength ϵ_*c*_ from 0.1 V nm^–1^ to 3.5 V nm^–1^ with a fixed cavity frequency of 3.9 eV. All simulations are performed with and without the approximate DSE terms (see eq 9) and the CBO dynamics is compared with the EJCM simulations. For the sake of clarity, we focus on the initial 15 fs of the dynamics. In Figure 3, all results are summarized.

The temporal evolution of the population in the ground state obtained using CBO with DSE terms is depicted in Figure 3a. All simulations up to a cavity field strength of 2.0 V nm^–1^ show an increase of the *S*_0_^*a*^ population within the first 10 fs. As expected, the amount of population transferred scales with ϵ_*c*_ and reaches a maximum of ∼70% for 1.5 V nm^–1^. For larger cavity field strengths, the temporal profile is becoming more and more structured and the maximum transferred population is becoming smaller. A third oscillation period of ∼4 fs appears and for the highest value of ϵ_*c*_, Rabi oscillations are visible after ∼10 fs (dark red features in Figure 3). In Figure 3b, the difference in the *S*_0_^*a*^ population with and without DSE terms included in the CBO dynamics are shown. As discussed in the literature,^19,20,26,48^ the DSE terms are important to correctly describe the light-matter interaction in a coupled molecular-cavity system in the dipole approximation. Here, neglecting DSE affects the population dynamics and the observed cavity-induced population transfer is overestimated. With increasing ϵ_*c*_, the effect of the DSE terms on the population transfer becomes stronger. For the highest cavity field strength of 3.5 V nm^–1^, the maximum difference is up to 0.15, or ∼25%–50% of the total transferred population. The last aspect investigated in the MgH^+^ system is the difference between the results obtained with the CBO ansatz and the EJCM ansatz. The difference in the *S*_0_^*a*^ population between both methods is shown in Figure 3c. As previously shown^27^ for the case of VSC, such an approach can give reliable results if the coupling is not too strong. This is also true in our case for ESC. Up to 1.0 V nm^–1^, the differences between CBO and EJCM are below 0.01. Similar to the observed effect of including DSE terms, increasing ϵ_*c*_ leads to a larger deviation of the CBO and EJCM results. For the highest investigated cavity field strength of 3.5 V nm^–1^, the maximum difference is 0.1, which corresponds to ∼15%–30% of the total transferred population. However, note that the overall trends observed in the CBO dynamics are comparable to the results of the EJCM simulations.

### IV.B LiF

**Figure 4:**
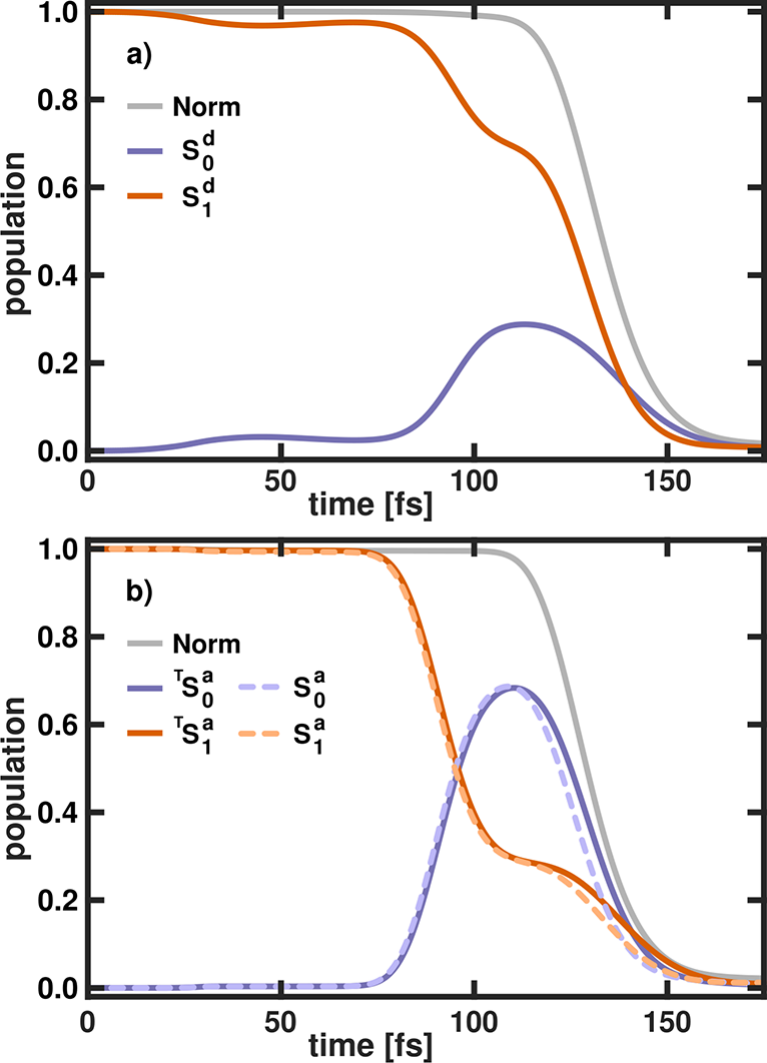
(a) Time-dependent diabatic populations in the two electronic states (*S*_0_^*d*^ and *S*_1_^*d*^) in LiF for ω_*c*_ = 3.9 eV and ϵ_*c*_ = 0.13 V nm^–1^ calculated with the CBO ansatz. (b) Time-dependent adiabatic populations in the two electronic states in LiF for the same cavity parameters obtained by transformation from the diabatic nuclear wave functions (^*T*^*S*_0_^*a*^ and ^*T*^*S*_1_^*a*^, solid lines) and directly from the adiabatic dynamics (*S*_0_^*a*^ and *S*_1_^*a*^, lighter dashed lines). The norm (total population) is depicted as a gray line in both panels.

The second system, LiF coupled to a cavity, makes it possible to study a more complicated situation. Here, nonadiabatic processes are already present in the bare molecular system, caused by an avoided crossing at a bond length of ∼13 a.u. (see Figure 1b). This situation occurs in diatomic molecular systems, as the conditions of a CoIn cannot be fulfilled with only one internal degree of freedom.^51−53^ When the diatomic molecule is coupled to the quantized vacuum field of a cavity, the electromagnetic field becomes an additional degree of freedom. The resulting coupled system is two-dimensional and a light-induced or cavity-induced CoIn can be formed.^23,75−77^ Since treating CoIns in the adiabatic representation can lead to numerical difficulties, because of strongly localized and diverging NACEs, the diabatic representation is often used in bare molecular systems.^51−54^ In our CBO simulations of LiF, we encounter the expected numerical instabilities in the adiabatic representation for a cavity field strength larger than 0.13 V nm^–1^. To test the validity of the adiabatic to diabatic transformation within the CBO ansatz, we compare the population dynamics of the coupled LiF system for both the adiabatic representation and the diabatic representation. The cavity parameter ω_*c*_ = 3.9 eV and ϵ_*c*_ = 0.13 V nm^–1^ are chosen to give stable adiabatic propagation. The results are shown in Figure 4.

The temporal evolution of the diabatic populations in the *S*_0_^*d*^ and *S*_1_^*d*^ state as well as the norm (total population) is depicted in Figure 4a. The corresponding adiabatic population dynamics is shown in Figure 4b. The solid lines represent the populations (^*T*^*S*_0_^*a*^ and ^*T*^*S*_1_^*a*^) obtained by the back transformation of the diabatic nuclear wave functions. The lighter dashed lines represent the populations (*S*_0_^*a*^ and *S*_1_^*a*^) obtained directly from the adiabatic dynamics. After ∼25 fs, a first, rather weak population transfer in the diabatic dynamics can be observed. Even though the wave packet is in a region where the cavity is resonant, the chosen cavity field strength is weak and the transfer can be attributed mostly to the bare molecular diabatic coupling. This is confirmed by the adiabatic population dynamics (see Figure 4b), where almost no transfer is observed at ∼25 fs. The diabatic crossing region (avoided crossing in the adiabatic representation) is reached after 90 fs and the diabatic ground state population *S*_0_^*d*^ increases to 30%. Simultaneously, the population of the ground state in the adiabatic representation increases to 70%. Within the next 30 fs, the first parts of the wave packet reach the edge of the grid and are absorbed by the PML. Until 175 fs, almost all of the wave packet is absorbed, which, in our model, is equivalent to a complete dissociation of LiF. Overall, the direct adiabatic population dynamics and the transformed diabatic dynamics are in a very good agreement. Only after the avoided crossing region (∼120 fs), the population decay due to PML is faster, by ∼1.5 fs. Since the simulation in the diabatic representation is numerically stable and gives the same results, it is used in the following discussions of the coupled LiF system.

**Figure 5:**
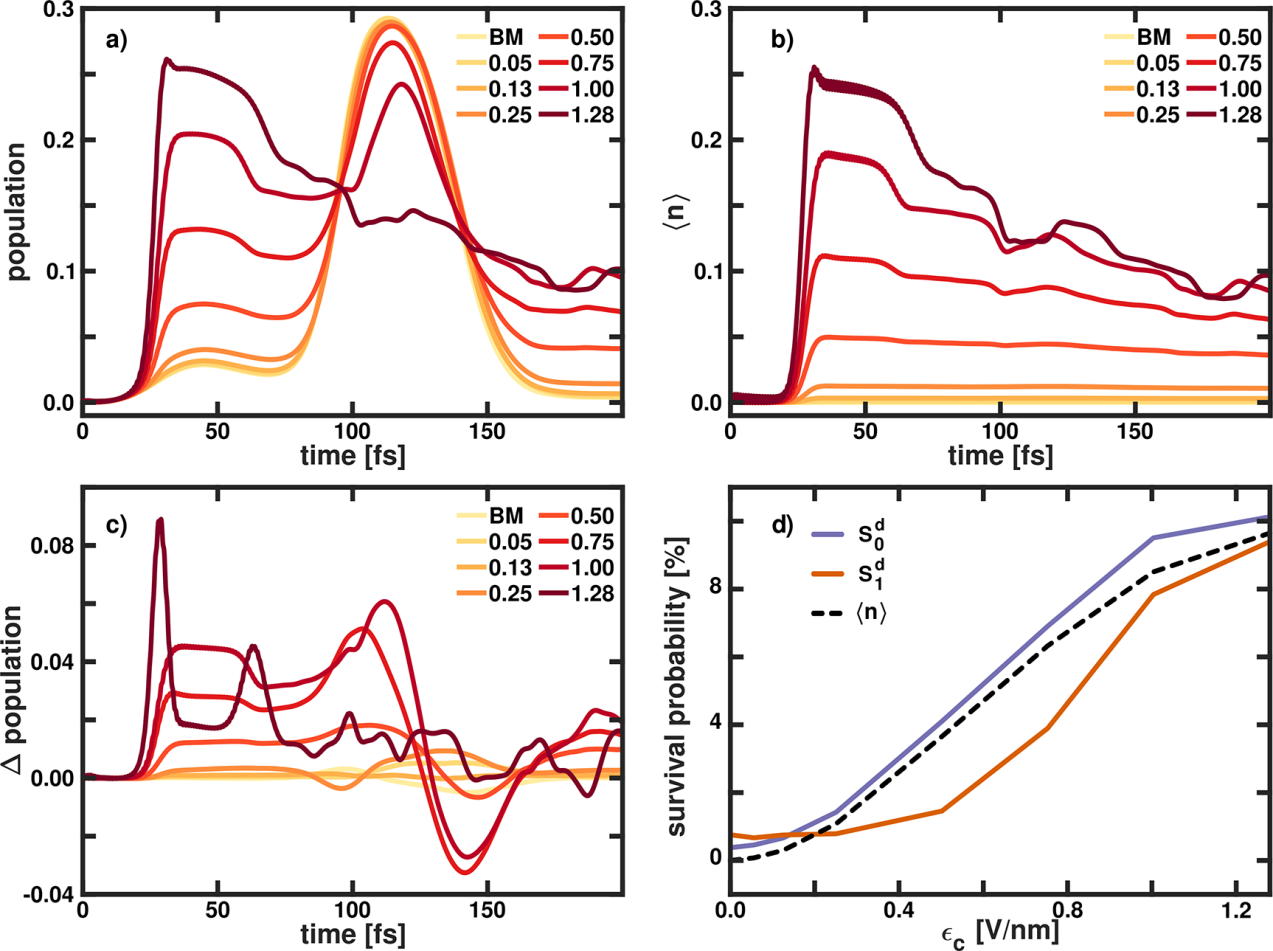
(a) Time-dependent diabatic population in the ground state *S*_0_^*d*^ of LiF obtained using CBO with DSE terms. (b) Time-dependent expectation value of the photon number ⟨*n*⟩. (c) Difference in the *S*_0_^*d*^ population between the CBO dynamics and the EJCM dynamics both with DSE terms included. All simulation were performed with ω_*c*_ = 4.0 eV, and the cavity field strength ϵ_*c*_ was increased from 0.0 V nm^–1^ (BM) to 1.28 V nm^–1^ (from lightest color to darkest color). (d) Survival probability in the *S*_0_^*d*^ state (violet) and *S*_1_^*d*^ state (orange), as well as the remaining photon number (dotted black line), as a function of ϵ_*c*_ for the CBO simulations. The survival probability is defined as the percentage of the population in the states at the end of simulation time.

To elucidate the effect of the coupling strength on the dissociation dynamics of LiF, ϵ_*c*_ is varied from 0.05 V nm^–1^ to 1.28 V nm^–1^ while ω_*c*_ is kept fixed at 4.0 eV. The results can be found in Figure 5. The expectation value of the photon number ⟨*n*⟩ and the population of the *S*_0_^*d*^ state in LiF are shown in Figures 5a and 5b, respectively. The differences in the *S*_0_^*d*^ population between the CBO dynamics and the EJCM dynamics are shown in Figure 5c. To describe the influence of the cavity on the dissociation, the survival probability as a function of ϵ_*c*_ is plotted in Figure 5d. We define the survival probability as the percentage of population in the *S*_0_^*d*^ state and in the *S*_1_^*d*^ state at the end of the simulation time. Additionally, the remaining photon number ⟨*n*⟩ is given as a percentage of one photon.

The temporal evolution of the ground-state population (Figure 5a) can be divided into three time intervals. The first interval covers the initial 80 fs of the dynamics and is characterized by the cavity being resonant with the electronic transition and a weak diabatic coupling in the bare molecule. For cavity field strengths of <0.13 V nm^–1^, the population transfer is mostly determined by the bare diabatic coupling. However, as ϵ_*c*_ increases, the cavity-induced population transfer becomes increasingly dominant. For the largest value of 1.28 V nm^–1^, ∼25% of the population is transferred to the *S*_0_^*d*^ state by the cavity interaction. In the subsequent interval (80–150 fs), the wave packet reaches the diabatic crossing region, and up to a field strength of 0.75 V nm^–1^, the temporal evolution of the ground-state population is very similar to that of the bare molecular system. For larger ϵ_*c*_ values, less population is transferred in this interval, indicating that, for stronger cavity coupling, the dynamics at the diabatic crossing region is changed. In the last interval, dissociation occurs. With increasing cavity field strength, more population is retained in the *S*_0_^*d*^ state. Up to a field strength of 0.75 V nm^–1^ the temporal evolution of the population is nearly constant after 170 fs. The time-dependent expectation value of the photon number ⟨*n*⟩ shown in Figure 5b complements the interpretation of the coupled dynamics. After ∼25 fs, the cavity is resonant with the electronic transition and ⟨*n*⟩ increases sharply. The maximum value of the photon number achieved depends on the magnitude of the coupling strength. After the initial rise, the temporal evolution of ⟨*n*⟩ can be separated into two regimes. Below 0.75 V nm^–1^, ⟨*n*⟩ stays approximately constant for the remaining simulation time. For larger ϵ_*c*_ values, the photon number decreases with time and the slope becomes steeper as the cavity field strength increases. Interestingly, the diabatic crossing region (80–150 fs) has no influence on ⟨*n*⟩. However, at all other times, the temporal evolution of ⟨*n*⟩ closely resembles the dynamics of the population in the ground state. In contrast to MgH^+^, the expectation value of the photon number ⟨*n*⟩ is not equivalent to the population of the *S*_0_^*d*^ state. In our ansatz, population is transferred between the two cPESs, because of nonadiabatic coupling elements naturally present in the molecule or induced by the cavity interaction. However, only the later ones lead to changes in the photon number. Since, for LiF, both pathways are possible, the population and ⟨*n*⟩ are not identical. A comparison of the population of the ground state obtained with the CBO ansatz and the EJCM ansatz yields similar results as in the MgH^+^ case. The differences between CBO and EJCM also increase as the coupling strength increases. For ϵ_*c*_ < 0.75 V nm^–1^, the main differences occur in the diabatic crossing region. These differences are also present for larger cavity field strengths, but the discrepancy between both methods becomes larger in the region when the cavity is resonant with the electronic transition. In Figure 5d, the survival probabilities in the *S*_0_^*d*^ state (violet) and the *S*_1_^*d*^ state (orange) and the corresponding photon number as a function of ϵ_*c*_ are shown. The remaining population in the ground state *S*_0_^*d*^, and the corresponding photon number are nearly identical and increase with increasing field strengths. The *S*_1_^*d*^ population remains nearly constant below 0.5 V nm^–1^ and increases significantly for higher values. For the highest field strength tested, the remaining populations is almost equally distributed in the *S*_1_^*d*^ state and the *S*_0_^*d*^ state. Increasing the cavity field strength suppresses the dissociation process in LiF as the survival probability increases from ∼1% to 20% overall. The different behavior of the population in the *S*_1_^*d*^ state and the *S*_0_^*d*^ state indicates that there are two effects active. The first one is due to the cavity being resonant with the electronic transition. In this situation, the interaction enables a decay channel in the bound ground state *S*_0_^*d*^, and simultaneously a photon is created in the cavity. Therefore, ⟨*n*⟩ and the *S*_0_^*d*^ population are very similar and the transferred wave packet is trapped. The efficiency of this process is getting larger with increasing ϵ_*c*_. For higher values of ϵ_*c*_, the diabatic crossing region is getting more deformed by the interaction with the cavity. This deformation reduces dissociation and also weakens the transfer of the population to the *S*_0_^*d*^ state, leading to an increase of the remaining population in the *S*_1_^*d*^ state. For larger coupling strengths, the Rabi splitting increases and the population transfer is suppressed.^18,76^

**Figure 6:**
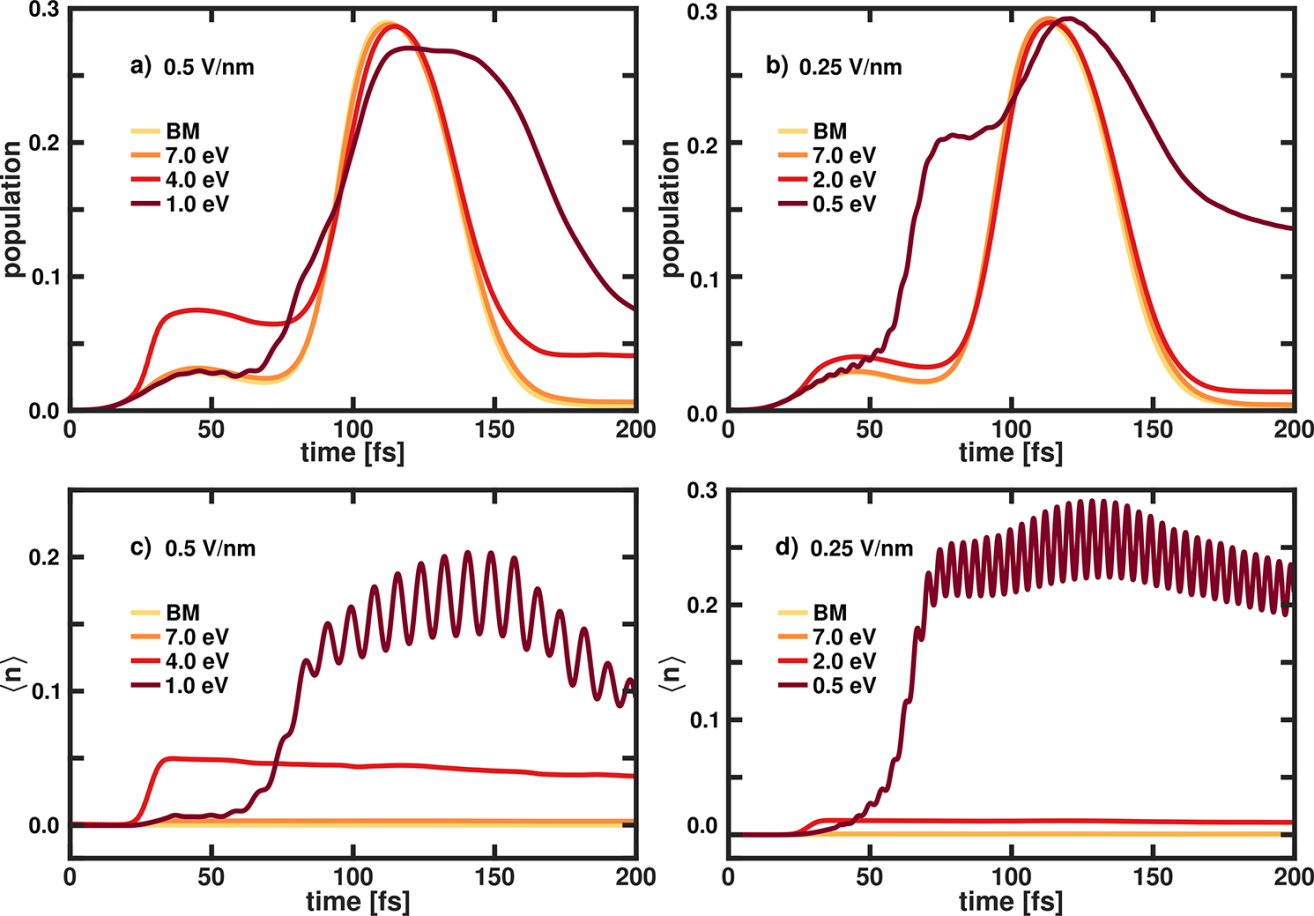
Time-dependent diabatic population in the ground state *S*_0_^*d*^ of LiF (top row) and expectation value of the photon number ⟨*n*⟩ (bottom row) for different cavity frequencies ω_*c*_ (decreasing from lightest color to darkest color) for two fixed cavity field strengths: (a, c) ϵ_*c*_ = 0.50 V nm^–1^ and (b, d) ϵ_*c*_ = 0.25 V nm^–1^. The lightest color indicates the bare molecule (BM).

In addition to the strength of the cavity coupling, the chosen frequency of the cavity ω_*c*_ can also affect the dynamics. By varying ω_*c*_, different regions of the bare molecular PESs become resonant with the cavity mode. For the coupled LiF system, we have considered different cavity frequencies that range from coupling near the FC region to coupling close to the avoided crossing, covering the range from 0.5 eV to 7.0 eV (see green lines in Figure 1b). As a result, we cover a pure ESC regime, as well as a mixed VSC and ESC regime. Figure 6 shows the ground-state population and the respective photon number ⟨*n*⟩ for two different field strengths (0.50 V nm^–1^ (Figures 6a and 6c) and 0.25 V nm^–1^ (Figures 6b and 6d)) for varying ω_*c*_.

When the cavity is resonant with the FC region, the temporal evolution of the population is marginally changed, compared to the bare molecule (BM), independent of the chosen cavity field strength, and the photon number is close to zero. For the three other cavity frequencies, the dynamics is affected more strongly by the interaction with the cavity. The smaller the cavity frequency, the later within the dynamics the resonance region is reached by the wave packet. This can be seen from the temporal shifted increase in ⟨*n*⟩ (see Figures 6c and 6d). In both the 0.5 eV case and the 4.0 eV case, the interaction between the molecule and the cavity is stronger, compared to the 7.0 eV case for the same cavity field strength. Rabi oscillations are clearly visible in the temporal evolution of ⟨*n*⟩ for the two smallest frequencies. The population dynamics is strongly modified for a cavity frequencies of 0.5 and 4.0 eV. Moreover, the survival probability after 200 fs is larger for low cavity frequencies. The increasing transition and permanent dipole moments close to the avoided crossing region in LiF may explain this behavior.

**Figure 7:**
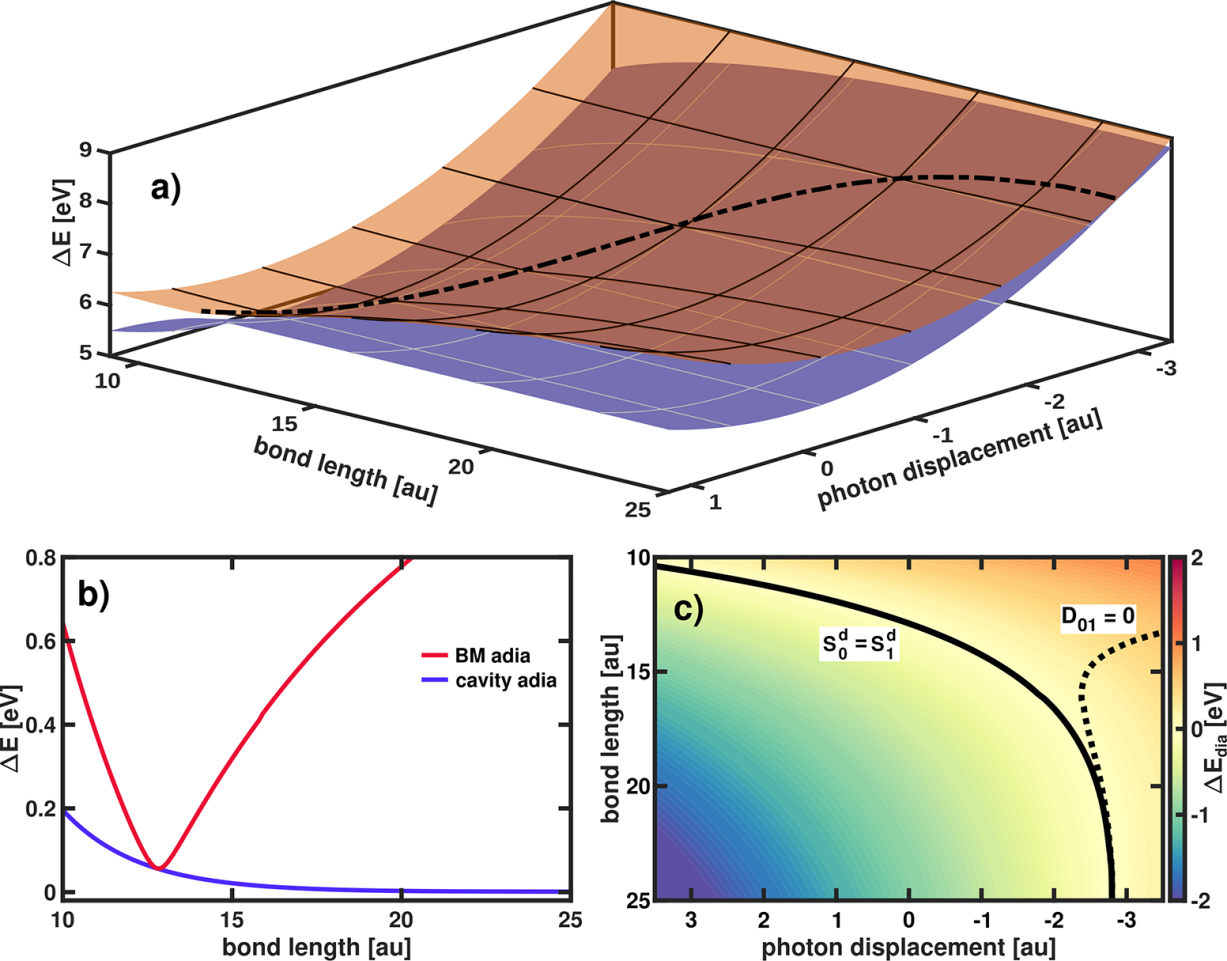
(a) Adiabatic PESs of the avoided crossing region of LiF coupled to cavity with ω_*c*_ = 4.0 eV and ϵ_*c*_ = 0.50 V nm^–1^. The black dotted line marks the seam of minimal energy gaps. (b) Minimal adiabatic energy gap Δ*E* along the bond length for the bare molecule (BM) (red) and the cavity coupled system (blue). (c) Energy difference Δ*E*_dia_ between the diabatic states of LiF coupled to a cavity. The black solid line indicates the crossing of the diabatic states and the dotted line marks the zero crossing of the diabatic coupling elements *D*_01_.

The CBO ansatz allows us to describe light-matter interactions and intrinsic nonadiabatic couplings on the same footing. Therefore, the ansatz could be a good choice to study cavity-induced or modified CoIns.^75−77^ We have shown that the cavity interaction modifies the nonadiabatic dynamics in LiF. In this last section, we thereby examine to what extent a cavity induced or modified CoIn is present. For ω_*c*_ = 4.0 eV and ϵ_*c*_ = 0.50 V nm^–1^, the adiabatic and diabatic PESs and couplings are calculated for a bond length between 10 a.u. and 25 a.u. and a photon displacement coordinate from −5 a.u. to 5 a.u. (shown in Figure 7).

The adiabatic CBO surfaces of *S*_0_^*a*^ and *S*_1_^*a*^ are shown in Figure 7a and the minimal adiabatic energy gap, as a function of the bond length, is plotted in Figure 7b. The cavity interaction transforms the avoided crossing point in the BM into a seam of minimal energy gaps in the adiabatic representation. Along this seam, the adiabatic energy difference becomes smaller as the bond length increases. Simultaneously, as the bond length increases, the photon displacement coordinate tends to a finite value (in the given example, −2.76 a.u.). For any given bond length, the BM energy gap is larger, compared to the minimal energy gap in the cavity-coupled system. To shed more light on this interesting situation, the corresponding diabatic energy difference of *S*_0_^*d*^ and *S*_1_^*d*^ is depicted in Figure 7c. The solid black line describes the diabatic crossing seam (*S*_0_^*d*^ = *S*_1_^*d*^) and the black dotted line describes the seam when the diabatic coupling element *D*_01_ is equal to zero. The diabatic crossing seam and the *D*_01_ = 0 seam are approaching each other and seem to coincide in the limit of further increasing bond length. Returning to the opening question of whether a cavity induced or a modified CoIn is formed in LiF, a simple answer cannot be given. Clearly, the entire avoided crossing region is strongly altered by the interaction with the cavity and the energy gap between the states is decreasing. For longer bond lengths, a point of degeneracy appears to be formed. However, this CoIn-like structure probably plays a subordinate role in the dissociation, since it is energetically not accessible. In addition, the position of this degeneracy (beyond a bond length of 25 a.u.) raises the question whether the system can still be described as a single molecule coupled to a cavity.

## V Conclusion

We have demonstrated how to perform nonadiabatic wave packet dynamics in the framework of the Cavity-Born-Oppenheimer (CBO) approximation. By implementing a modified electronic Hamiltonian for the CASSCF method and for the MRCI method, we are able to calculate highly accurate energies and nonadiabatic couplings within the CBO. Using the obtained cPESs and the corresponding couplings, the excited-state dynamics of the coupled cavity-molecular system was simulated by means of nonadiabatic wave packet dynamics. The influence of the cavity on the molecular system is included in the cPESs (VSC effects) and in the nonadiabatic coupling elements (ESC effects). In this approach, the latter ones are formally equivalent to the bare molecular couplings.

As the first test case, we have studied the dynamics of the bound state of MgH^+^. In this system, the cavity-induced coupling is the only channel of population transfer between the ground state and the excited state. The resulting temporal evolution of the population and the photon number directly reflects the molecular motion in both states involved. The CBO results are in quantitative agreement with the EJCM simulations for a cavity field strength of 1.0 V nm^–1^. Moreover, for larger coupling strengths, the overall trends are still comparable. In accordance with the literature,^19,20,26,48^ we observed that neglecting the DSE terms leads to an overestimation of the effects induced by the cavity. In the second example, we have focused on the photodissociation of LiF coupled to a cavity mode. LiF exhibits an intrinsic avoided crossing, which is strongly modified by cavity interaction. To obtain a numerically stable dynamics simulation, we have performed the calculation in the diabatic representation. With increasing cavity field strength, the survival probabilities increase due to two effects. First, the interaction with the cavity field directly transfers the population to the bound ground state and creates a photon in the cavity. Second, the intrinsic crossing region is deformed, trapping the molecule in the excited state. When the characteristic frequency of the cavity is decreased, the dissociation dynamics is influenced even more strongly and the survival probability also increases.

We have shown that it is possible to perform nonadiabatic wave packet dynamics within the framework of CBO. This ansatz allows us to describe the dynamics in a strongly coupled molecular-cavity system in an accurate way based on ab initio cPESs and nonadiabatic/diabatic couplings. As discussed in the LiF case, the CBO ansatz could be a good choice for studying cavity-induced or modified CoIns.^75−77^ In this work, we have restricted ourselves to a single molecule coupled to a single-cavity mode. Note that for a reasonable description of VSC or ESC in an ensemble of molecules, as it would appear in an experiment, our ansatz must be extended to include the interaction within the ensemble and multiple cavity modes. Such an ensemble can, in principle, be one or more molecules interacting with the environment, such as a solvent. However, also other factors that have pronounced impacts, such as energy loss and dephasing effects, should be investigated in future work.
